# Optical Coherence Tomography Angiography in Pediatric Myopia and Amblyopia: A Review of Clinical Applications, Microvascular Biomarkers, and Treatment Monitoring

**DOI:** 10.2174/0115734056490478260821115658

**Published:** 2026-08-27

**Authors:** Jiajun Wang, Daohuan Kang, Huixia Hua, Caiping Shi, Yiguo Pan, Yongwei Guo, Wen Sun

**Affiliations:** 1 Department of Ophthalmology, Children’s Hospital, Zhejiang University School of Medicine, National Clinical Research Center For Children and Adolescents’ Health and Diseases, No.3333, Binsheng road, Binjiang District, Hangzhou, 310003, China; 2 Eye Center, The Second Affiliated Hospital, School of Medicine, Zhejiang University, Zhejiang Provincial Key Laboratory of Ophthalmology, Zhejiang Provincial Clinical Research Center for Eye Diseases, Zhejiang Provincial Engineering Institute on Eye Diseases, Hangzhou, Zhejiang, China

**Keywords:** Optical Coherence Tomography Angiography (OCTA), Pediatrics, Myopia, Amblyopia, Foveal Avascular Zone (FAZ), Vessel Density (VD), Neuro-ophthalmic conditions, Diabetic retinopathy

## Abstract

Optical Coherence Tomography Angiography (OCTA) provides depth-resolved imaging of the retinal vascular system without invasion. Its rapid and non-invasive characteristics make it a promising tool for pediatric retinal imaging. Pediatric myopia and amblyopia can affect visual development and long-term visual prognosis. Early identification and dynamic monitoring are of great clinical significance. This paper retrieved English literature published from January 2006 to May 2026 in PubMed and Web of Science, and reviewed the application of OCTA in pediatric myopia and amblyopia. Existing studies show that pediatric myopia is characterized by decreased retinal vascular density (VD), thinner choroidal thickness (CT), and changes in choroidal perfusion parameters; amblyopia is mainly manifested as decreased retinal VD, but results for the foveal avascular zone (FAZ) and choroidal-related indicators are inconsistent. At present, there are still some deficiencies in the research, such as limited sample size, non-uniform equipment and scanning protocols, and lack of long-term follow-up. OCTA is expected to become an important auxiliary tool for the diagnosis, evaluation, and monitoring of treatment for childhood myopia and amblyopia.

## 1 INTRODUCTION

Optical Coherence Tomography Angiography (OCTA) is a non-invasive technique for analyzing retinal microvasculature *in vivo*. It is based on Optical Coherence Tomography (OCT), which uses infrared light to capture signals reflected from retinal tissues. OCTA detects variations between repeated OCT B-scans caused by erythrocyte movement within the vascular system [1, 2]. This technique enables assessment of the Foveal Avascular Zone (FAZ), Vessel Density (VD), and retinal perfusion without the potential complications associated with dye injection [3, 4]. The Superficial Capillary Plexus (SCP) on OCTA consists of vessels in the ganglion cell layer and the nerve fiber layer, whereas the Deep Capillary Plexus (DCP) encompasses the deep inner and intermediate retinal vasculature [5].

OCTA has been widely used in adults to investigate retinal and choroidal circulation in different vascular conditions and neuro-ophthalmic conditions, such as diabetic retinopathy [6], retinal vein occlusion [7], optic neuropathies [8], age-related macular degeneration [9, 10], glaucoma [11, 12], and inherited retinal disorders [13].

Because OCTA is less invasive and faster to acquire than dye-based angiography, it has improved our understanding of retinal and choroidal vasculature in children [14]. OCTA has been employed to evaluate the pediatric retina and retinal development in inherited retinal degenerations, retinal vascular diseases, tumors, trauma, inflammatory conditions, retinopathy of prematurity, Coats disease, diabetic retinopathy, and pediatric glaucoma [14-16]. However, OCTA and traditional dye-based angiography do not have identical roles in pediatric ocular diseases. Dye-based angiography can directly demonstrate vascular leakage and remains more suitable for assessing peripheral non-perfusion, leakage, neovascular activity, and treatment boundaries. Therefore, OCTA should currently be regarded as a complementary modality rather than a complete replacement for dye-based angiography in many pediatric retinal vascular diseases.

Due to the non-invasive and reproducible nature of OCTA, recently, there has been an increasing focus on the application of OCTA in the research of myopia and amblyopia. The advantage of existing studies lies in the ability to non-invasively and quantitatively analyze the microvascular parameters of the retina and choroid, providing new imaging evidence. Nevertheless, most current studies are single-center, small-sample, and cross-sectional, lacking long-term follow-up and a standardized reference database for children. Moreover, there is a lack of systematic comparative summaries for these two types of diseases. In particular, there is significant heterogeneity among different studies in terms of disease subtypes, age ranges, OCTA parameters, *etc*., which limits the translation of relevant indicators into clinical practice. Therefore, it is necessary to summarize the application of OCTA in pediatric myopia and amblyopia, and clarify the advantages, limitations, and future development directions of existing studies. This review aims to summarize the relevant research progress, analyze the clinical significance of major OCTA parameters, and explore its potential value in the early assessment, treatment monitoring, and pathogenesis research of pediatric myopia and amblyopia.

## 2 METHODOLOGY

Relevant literature was searched on PubMed and Web of Science using the term “optical coherence tomography angiography” OR “OCTA” combined with “pediatric, children, or adolescent” and paired with a specific disease term such as “myopia” and “amblyopia”. The review covered papers published from January 2006 to may 2026. The criteria for literature inclusion were as follows: (I) OCTA findings in pediatric or adolescent patients with myopia or amblyopia as the topic of study, (II) articles, meta-analyses, and reviews, and (III) English-language literature. Meanwhile, the exclusion criteria were as follows: (I) editorial material, letters, editorials, and meeting abstracts; (II) not written in English; and (III) off-topic studies, including those that did not focus on OCTA data in myopia or amblyopia. The process is presented in Fig. (**1**). To strengthen the evidence base of this review, we further conducted a structured evaluation of the richness, diversity, information content, and reliability of the included literature. The specific evaluation contents include the types of diseases studied, the age range of the study subjects, the OCTA devices and scanning protocols, the analysis levels, the main quantitative parameters, and the consistency of the research results. Due to the significant differences among different studies in terms of OCTA devices, scanning ranges, segmentation algorithms, AL or image magnification corrections, disease subtypes, and the definitions of outcome indicators, it is not suitable to conduct a unified quantitative meta-analysis of all the results. This review primarily uses structured narrative synthesis and analysis of the consistency of results to summarize the existing evidence.

## 3 OCTA IN MYOPIA

Myopia, defined as a refractive error caused by excessive axial elongation of the eye, is an escalating global public health concern, affecting over two billion people [17, 18]. Any degree of myopia raises the risk of eye diseases, and this risk increases markedly at high myopia levels [18]. Early prevention of myopia and monitoring its progression are crucial. OCTA reveals vascular and structural changes in the retina and choroid and thus provides novel indicators for tracking myopia.

### 3.1 OCTA Findings in Choroidal Parameters

OCTA can quantify choroidal parameters such as Choroidal Thickness (CT), Choroidal Vascularity Index (CVI), Choroidal Vascularity Volume (CVV), and choriocapillaris flow area (CC) to assess medium- to large-sized choroidal vessels and the choriocapillaris. Representative OCTA images (Fig. **2**) [19]. Studies indicated that both medium and large choroidal vessels, as well as choroidal capillaries, may contribute to the development of myopia [20, 21]. Using OCTA, Jiang *et al*. [22] reported a thinner macular choroid and a reduced number of flow voids in the nasal macular area of myopic eyes. Ye *et al*. [23] showed that axial length to corneal curvature radius ratio (AL/CR) correlated with choroidal blood perfusion. Zhou *et al*. [24] observed that the size and density of choriocapillary flow deficit (CCFD) increased in moderately myopic children aged 8-16 years. Cao *et al*. [25] found that increased AL was significantly related to decreased CVV, CT, and choroidal stromal volume (CSV). Overall, these findings highlight the need for careful attention to alterations in choroid microcirculation in myopic children.

### 3.2 OCTA Findings in Retinal Parameters

OCTA can provide retinal parameters such as retinal thickness (RT), VD, and FAZ circularity, which serve as novel biomarkers to support the diagnosis and management of myopia. Those metrics offer valuable insights for early detection and monitoring of retinal changes associated with myopia [26]. Optical Coherence Tomographic Angiography scans of the macula and optic nerve (Fig. **3**) [27]. Pourbagherkhah *et al*. [28] found that VD in SCP decreased with increasing myopia severity, whereas FAZ remained unchanged. High myopia correlated with reduced VD, notably in the foveal and paramacular regions of SCP and DCP. The VD in the macular fovea was higher in mild myopia compared to high myopia, whereas the VD of SCP in the paramacular fovea was comparable between mild and high myopia groups. Axial elongation was a key factor underlying retinal vascular changes in myopia. The results emphasize the progressive influence of myopia severity on retinal microvasculature. Therefore, regular retinal examinations are crucial for individuals with severe myopia to track vascular alterations longitudinally. Leng *et al*. [29] examined 69 subjects and reported that VD decreased in SCP but remained unchanged in DCP in myopic patients (Fig. **4**). Gołębiewska *et al*. [30] reached a consistent conclusion. However, Cao *et al*. [25] reported that VD decreased in DCP but remained unchanged in SCP. Shuaib *et al*. [31] further examined patients with high myopia and demonstrated that VD decreased in long-axial eyes, which is consistent with the research results of Mu *et al.* [32]. Zhu *et al*. [33] reported that only VD of DCP decreased in myopia. However, the age range included in their study was relatively narrow, covering only adolescents aged 14 to 18 years old.

Gołębiewska *et al*. [30] reported an enlargement of the FAZ area in myopic eyes. Many studies reached a consistent conclusion [34-37]. However, no changes were observed in the FAZ in other studies [29, 31, 38]. In the research on pediatric myopia, the inconsistent results related to the FAZ may be associated with various methodological and biological factors. First, the FAZ area is affected by age, axial length, refractive status, and the degree of macular development. Different age ranges and myopia severities of the children included in various studies may lead to differences in the results. Children with high myopia or long axial lengths may exhibit an enlarged FAZ due to mechanical traction in the macular area, retinal thinning, and capillary rarefaction. In contrast, the changes in macular microvasculature in children with low - to moderate myopia or younger age may not be obvious, so the FAZ changes are not significant. Second, changes in axial length can cause magnification errors in OCTA images. If the AL or image magnification is not corrected, the FAZ area and VD may be overestimated or underestimated. Additionally, differences in OCTA equipment, scanning range, image quality, projection artifact correction, and automatic segmentation algorithms can also affect the identification of FAZ boundaries and the quantitative results. Therefore, whether the FAZ is enlarged should not be simply regarded as contradictory but should be comprehensively interpreted in combination with age, axial length, myopia degree, artifact correction, and imaging methods.

### 3.3 OCTA Findings in High Myopia

High myopia, a prevalent cause of vision impairment, is on the rise worldwide [17]. It is associated with various pathological conditions such as posterior staphyloma, epiretinal membrane, Myopic Macular Schisis (MMS), macular hole, chorioretinal dystrophy, and retinal detachment [39, 40]. High myopia causes numerous complications and substantial harm; thus, many studies have focused on it. In a study by Wu *et al* [41], using optical coherence tomography (OCT) and OCTA, a reduction in retinal and choroidal thickness, as well as a reduction in retinal VD in individuals with high myopia. The retinal microvascular network may exhibit a compensatory response to the hypoxic conditions associated with high myopia. Lin *et al*. [42] conducted a prospective study using OCTA and found that the loss rate of VD in the DCP was significantly faster in the highly myopic group and was associated with the baseline AL. In eyes with a longer baseline AL, the VD in the outer ring decreased more rapidly. These results provide new insight into the process of high myopia, and support macular VD as a contributor to the mechanisms underlying high myopia–related complications. Tang *et al*. [43] detected the OCTA changes in the optic nerve head (ONH) microcirculation in high myopia. They revealed that in patients with high myopia, neither Intrapapillary Vascular Density (IVD) nor peripapillary vascular density (PVD) exhibited significant changes with age or the increase of spherical equivalent (SE). However, as AL increased, IVD remained stable, while PVD decreased, particularly in the temporal regions. Furthermore, PVD demonstrated a negative correlation with AL. In young individuals, there was no noteworthy association observed between SE, age, and vascular density of the ONH. It is advisable for ophthalmologists and myopic patients to focus on the extension of AL rather than solely on refractive changes. Li *et al*. [44] observed a decrease in retinal microvascular density in patients with high myopia using OCTA. The study suggested that alterations in the vascular system might precede structural changes to impact macular function during the early stages of high myopia. Quantitative analysis of retinal microvascular could improve comprehension of the pathophysiological processes involved in myopia, potentially enabling early identification and prevention of myopic retinopathy.

### 3.4 Evaluation of Myopia Prevention and Control Measures by OCTA

The rising prevalence of myopia is attributed to near-sight work, the widespread use of electronic products, and decreased outdoor activities. To address this issue, interventions are crucial to impede myopia progression. Current approaches to managing childhood myopia concentrate on enhancing environmental factors, using medications, and employing optical aids. These aids, including multifocal soft contact lenses (SCL), orthokeratology (OK), and peripheral defocus spectacles, are frequently utilized to decelerate myopia advancement in children [45]. Therefore, additional investigation and study on preventing and managing myopia are imperative.

Abnormalities in the retinal microcirculation have been observed in many ocular and systemic diseases. During initial disease phases, alterations in retinal microcirculation can offer diagnostic indications. OCTA enables non-invasive visualization of the retinal and choroidal vessels, generates data on anatomical features and perfusion status, and quantifies the vascular perfusion density of different retinal layers. OCTA was used to assess the safety of atropine, low-level red-light therapy (LLLT), and orthokeratology. Tsai *et al*. [46] followed children receiving 0.125% atropine for one year, and observed no significant persistent changes throughout follow-up, supporting the safety of this regimen. LLLT was reported to be safe [47, 48], because the therapy did not produce abnormalities in macular microcirculation, fundus structure, or visual function. Longitudinal follow-up was conducted on adolescents with orthokeratology; OCTA showed increased macular VD in both the SCP and DCP, indicating that brief OK use can increase retinal VD, with a high level of safety during standard wear [38]. In the future, studies on the safety and efficacy of OCTA in the use of medications, multifocal soft contact lenses, and peripheral defocus glasses for myopia control can also be considered.

In summary, OCTA can be used to detect subtle changes, guide treatment strategies, ensure treatment safety, and monitor the effects of prevention and control measures. The above research demonstrates the potential of these novel imaging modalities in myopia. The OCTA parameters in myopia are summarized in Table **1**.

## 4 OCTA IN AMBLYOPIA

Amblyopia is a lasting decrease in vision caused by deficient cortical visual development resulting from atypical visual encounters during childhood. This neurodevelopmental condition lacks ocular pathology and is identified by diminished eyesight in one or both eyes, with an interocular visual gap of two lines or greater [49].

Previous studies have shown changes in the visual pathways, visual cortex, and retina in amblyopic eyes, while the presence of retinal microvascularization changes in these eyes is still uncertain [50-52].

### 4.1 OCTA Findings in Retinal Parameters

Early studies did not distinguish between different subtypes of amblyopia for research. Kim *et al*. [53] demonstrated that patch occlusion treatment led to an increased foveal VD of DCP in amblyopic eyes. The improvement in BCVA correlated with increased foveal VD of DCP. These results may help predict visual-acuity improvement following patch occlusion treatment. Errera *et al*. [54] included 64 eyes of 32 patients with unilateral amblyopia caused by strabismus, anisometropia, and unilateral cataract. A statistically significant reduction in VD of SCP was observed in 3×3mm^2^ and 6×6mm^2^ scans of amblyopic eyes. The FAZ shape in amblyopic eyes was less circular than in controls, suggesting irregular FAZ boundaries, which may indicate macular vascular impairment. Sobral *et al*. [55] included 52 eyes of 52 patients with unilateral amblyopia caused by strabismus and anisometropia, noting a significant reduction in macular VD of SCP, and a slight increase in the FAZ area of DCP. Huang *et al*. [56] analyzed 28 eyes of 14 children with unilateral amblyopia caused by strabismus and anisometropia. Unilateral amblyopic eyes exhibited notably reduced VD in the macula. Macular thickness, FAZ area, and optic disk VD were comparable across amblyopic eyes, fellow eyes, and control eyes. Yang *et al*. [57] found that amblyopia patients exhibited decreased foveal, parafoveal, and radial peripapillary capillary VD. The superficial and particularly deep FAZ were larger in amblyopia patients than the control group. Feng *et al*. [58] revealed that the VD was lower in certain areas of the SCP and DCP in amblyopic eyes, which was consistent with the previous research results (Fig. **5**) [27, 59]. The retinal microvascular alterations observed in amblyopic eyes may suggest anomalies or delayed maturation of the macular associated with insufficient visual stimulation. Animal studies have demonstrated that depriving one eye can result in reduced density of retinal ganglion cells [60] and a general thinning of the inner plexiform layer [61, 62]. Deprivation of vision may lead to a reduction in synaptic connections in the inner plexiform layer [63], linked to decreased activity of retinal ganglion cells [64]. Amblyopic patients show thinning of the ganglion cell layer and inner plexiform layer in OCT studies [65]. The reduced VD in amblyopic eyes may indicate lower oxygen and nutrient needs in the inner retina due to diminished ganglion cell number and activity [66]. However, some studies have not found significant changes in VD, which may be related to a small sample size, milder amblyopia, different treatment states, different scanning regions, or using the contralateral eye as a control. It is worth noting that the contralateral eye of patients with monocular amblyopia may also be affected by abnormal visual development. Therefore, using the contralateral eye as the sole control may underestimate the differences between the amblyopic eye and normal children. Regarding the FAZ, some studies have reported an enlarged or irregularly shaped FAZ, while others have shown no significant changes. This may be related to differences in amblyopia subtypes, refractive status, AL, macular development, image magnification correction, and OCTA segmentation algorithms. Therefore, current evidence suggests that amblyopia may be accompanied by changes in macular microvasculature, but the characteristics are not entirely consistent. In the future, stratified analysis according to amblyopia subtypes, age, refractive status, and treatment stages is still needed.

### 4.2 OCTA Findings in Choroidal Parameters

In recent years, with the development of three-dimensional quantitative analysis, OCTA research has expanded to more detailed choroidal and choroidal capillary parameters. Cao *et al*. [67] found that using OCTA in children with hyperopic anisometropic amblyopia, the CT, CC, and 3D-CVV of the amblyopic eye increased compared with the contralateral eye, and the 3D-CVI and 3D-CVV were higher than those of the normal control eye. It is suggested that there may be changes in choroidal vascular volume and vascular composition in hyperopic anisometropic amblyopia. Liu *et al*. [68] combined OCTA parameters and machine learning models to analyze adolescents with hyperopic refractive amblyopia and found that the choroidal thickness in the central and nasal sides of the amblyopic eye increased, further supporting the potential value of choroidal structural parameters in the identification of amblyopia. However, there are still differences in the latest research results. Li *et al*. [69] found in preschool children with hyperopic amblyopia that the 3D-CVI of foveal and parafoveal regions in amblyopic eyes decreased, CT thickened, while CVV showed no significant change. Karaca *et al*. [19] used SS-OCTA in children aged 6-14 with anisometropic amblyopia and performed axial length correlation amplification correction. They found that CVI decreased and CSV increased in some areas of the amblyopic eye, while there was no significant difference in CVV. Overall, existing studies suggest that amblyopia in children may be accompanied by microstructural differences in the choroid, but the conclusions of different studies are not entirely consistent. This difference may be related to the subtype of amblyopia, refractive status, age range, AL, scanning range, OCTA device, segmentation algorithm, magnification correction method, and the selection of two-dimensional or three-dimensional choroidal indicators. Therefore, a large number of prospective studies, including follow-up studies, need to be further conducted in the future to clarify the potential clinical significance of choroidal parameters in the diagnosis and treatment monitoring of amblyopia in children.

Recently, research on amblyopia has become more specialized, and researchers have conducted studies on the same subtype of amblyopia. Amblyopia is classified into anisometropic, strabismic, ametropic, and form-deprivation types. The following sections provide a detailed description of retinal parameters of amblyopia.

### 4.3 Anisometropic Amblyopia

Li *et al*. [70] reported that in children with anisometropic amblyopia, the macular and optic disc VD observed in OCTA was reduced, whereas the FAZ area was increased. These parameters tended to partially return to normal levels following amblyopia treatment. Therefore, OCTA is a promising device for the objective diagnosis and monitoring of amblyopia. Menacherry *et al*. [71] included 80 eyes of 40 children with unilateral anisometropia and discovered that the VD in amblyopia eyes decreased in both SCP and DCP. Doğuizi *et al*. [72] found that hyperopic anisometropic amblyopic eyes showed significantly reduced in foveal VD and VD in specific regions of SCP and DCP. No significant differences were observed in perimeter values or FAZ area between the amblyopic and fellow eyes. Hamurcu *et al*. [73] observed a notable reduction in VD of SCP of anisometropic amblyopic eyes, while no significant difference was found in the FAZ between amblyopic eyes and healthy eyes. Chung *et al*. [74] found that anisometropic amblyopic eyes exhibited no significant difference in the macular VD of the SCP between amblyopic and fellow eyes. However, these eyes had significantly reduced superficial FAZ. Newer three-dimensional OCTA analysis may provide more detailed vascular phenotyping; anisometropic amblyopic eyes showed reduced vessel volume density (VVD) and vessel skeleton density (VSD) [75]. Pekica *et al*. [76] analyzed 28 eyes of 14 children with unilateral anisometropic amblyopia, and their study findings indicated no statistically significant difference in the VD and FAZ of SCP, as well as in the full thickness of the central macula, between the amblyopic and non-amblyopic eyes in children diagnosed with unilateral anisometropic amblyopia. The findings of their study were consistent with the results of prior research [77]. However, the findings of their study were in opposition to those of prior research, which have consistently demonstrated the influence of amblyopia on vascular morphology [78-80]. The divergent outcomes of this study may be attributed to the limited sample size or the utilization of the contralateral eye as the control. Past literature suggested that unilateral amblyopia can indeed exert effects on the contralateral eye [81]. These differences in outcomes may also be attributed to the lack of magnification correction. Sampson *et al*. illustrated the effects of image magnification on VD and FAZ area [82].

### 4.4 Strabismic Amblyopia

Pujari *et al*. [83] showed the foveal VD of SCP in eyes with strabismic amblyopia was comparable to that in normal eyes in the 4.5×4.5mm^2^ scan. Yilmaz *et al*. [84] reported a significant reduction in the VD of both superficial and deep capillary plexuses in the 1-, 2-, and 3-mm^2^ regions measured by OCTA in patients with strabismic amblyopia. However, they found no significant difference in FAZ. Lonngi *et al*. [27] found that in the 6×6 mm^2^ measurement of the DCP, the VD of the amblyopic eyes decreased significantly. The differences in the OCTA examination results of children with amblyopia may be due to the lack of a uniform measurement method. When using 6×6 or 3×3mm^2^ scans, significant differences in the results have already been observed. Various OCTA systems utilize distinct software and algorithms, potentially contributing to discrepancies in retinal vascularization outcomes [5, 85-87].

### 4.5 Ametropic Amblyopia

Chung *et al*. [74] demonstrated that the bilateral ametropic amblyopia eyes exhibited a significantly reduced nasal VD of the SCP. Superficial FAZ areas and macular VD showed no difference between amblyopic eyes and healthy eyes. Rao *et al*. [88] found that the VD of hyperopic ametropic amblyopia patients decreased significantly; the VD showed a significant increase after treatment. The perimeter and circularity of FAZ exhibited notable differences between the groups. Following the 6-month treatment, the FAZ perimeter and area decreased, while circularity increased. These differences can be attributed to the variations in the measuring instruments used in the studies and the differences in the severity of amblyopia.

### 4.6 Form-deprivation Amblyopia

Simsek *et al*. [89] included children with ametropic, anisometropic, strabismic, and deprivation amblyopia and found that VD in both SCP and DCP were significantly lower in anisometropic, strabismic, and deprivation amblyopia than in ametropic amblyopia and healthy controls. This study provided direct OCTA data on form-deprivation amblyopia, although the deprivation subgroup remained relatively small. At present, there is scarce evidence of OCTA in children with form-deprivation amblyopia, which may be related to insufficient fixation and imaging quality caused by congenital cataracts, ptosis, corneal opacity, *etc*.

Overall, the non-invasiveness of OCTA and its ability to provide detailed information about the retinal vasculature make it appear to be a relevant and objective supplementary examination for diagnosing and monitoring amblyopia. The OCTA parameters in amblyopia are summarized in Table **2**.

## 5 BROADER IMPACT OF PEDIATRIC MYOPIA AND AMBLYOPIA ON OCULAR VASCULAR ARCHITECTURE

From the perspective of the development of pediatric ocular vascular structure, OCTA changes in myopia and amblyopia are not merely local vascular abnormalities but involve multi-level vascular changes in the retina, peripapillary and macular microcirculation, choroidal capillaries, and choroid. Myopia is mainly related to axial elongation and posterior pole mechanical traction, often manifested as decreased macular VD, lower DCP perfusion, thinner choroid, reduced choroidal blood flow parameters, and increased CCFD, suggesting that myopia - related remodeling is not limited to the inner retina but extends to the deep vascular supply area of the outer retina. Although amblyopia is considered a neurodevelopmental disorder without obvious ocular disease, OCTA studies have found reduced macular VD, while the changes in FAZ, choroidal capillaries, and choroidal parameters are inconsistent. Overall, myopia is more manifested as structural vascular remodeling related to AL, while amblyopia is more likely to reflect vascular changes related to abnormal visual development. Future studies should combine AL, refractive status, visual acuity level, treatment response, and long-term follow-up results to clarify the clinical value of these OCTA vascular indicators in the monitoring of children's eye diseases.

## 6 TECHNICAL LIMITATIONS, UNCERTAINTY, AND FUTURE TECHNOLOGICAL IMPROVEMENTS IN PEDIATRIC OCTA

Although OCTA has obvious advantages in pediatric ophthalmology, the further expansion of its clinical application still depends on the continuous improvement of imaging technology and image analysis methods. Pediatric patients have limited fixation ability, and eye movements and blinks are prone to occur during the examination. Motion artifacts are often important factors affecting the quality of pediatric OCTA images. Real-time eye movement tracking and motion correction techniques can reduce image misalignment and blood flow signal interruption caused by eye movements to a certain extent, thereby improving the stability of relevant parameter measurements [90]. Meanwhile, a higher A-scan rate and faster scanning protocol can shorten the image acquisition time and reduce the impact of motion during pediatric examinations. However, the scanning speed itself may also affect the OCTA vascular quantitative indicators. Therefore, a standardized protocol still needs to be established in the future to balance the improvement of acquisition speed and the comparability of parameters [91]. In addition, wide-field and ultra-wide-field OCTA can expand the imaging range, which is helpful for observing peripheral retinal vascular abnormalities, while handheld OCTA systems improve the feasibility of examinations for infants and young children [92-94]. Therefore, with the development of motion artifact suppression, scanning speed improvement, wide-field imaging, handheld devices, and artificial intelligence-assisted segmentation technology, the application scope of OCTA in pediatric myopia, amblyopia, and other pediatric eye diseases is expected to be further expanded.

## 7 INTEGRATED ARTIFICIAL INTELLIGENCE APPROACHES IN PEDIATRIC OCTA

An integrated artificial intelligence (AI) approach can further enhance the clinical value of OCTA in pediatric ophthalmology [95]. Deep learning models can automatically segment retinal vessels and FAZ in OCTA images, and further quantify a series of parameters such as the area, circumference, and irregularity index of FAZ, as well as the VD in the fovea centrula, paraventral fovea, and perioptic disc regions [96-99]. These automated measures can aid clinicians in report interpretation and reduce human error. This is particularly important for pediatric patients, as unstable fixation and motion artifacts may affect image quality.

In recent years, hybrid models combining convolutional neural networks (CNNS) and fuzzy logic have gradually attracted attention. CNN has a strong ability to automatically extract image features and can learn deep features such as retinal vascular course, capillary sparsity, FAZ boundary morphology, and local perfusion changes from OCTA images. Fuzzy logic, on the other hand, can handle common uncertainties and fuzziness in medical images, such as segmentation and interpretation biases caused by unstable fixation, motion artifacts, low image contrast, or unclear vascular boundaries in children's OCTA scans [100, 101]. Therefore, by combining the feature learning ability of CNN with the approximate reasoning ability of fuzzy logic, this hybrid AI framework can not only be used to assist in the early identification of microcirculation abnormalities related to myopia and amblyopia, but also integrate clinical variables such as axial length of the eye, refractive status, best corrected visual acuity and treatment history to guide individualized intervention for myopia control or amblyopia treatment. It is also used for longitudinal monitoring of treatment responses and can even form more individualized risk assessment and follow-up monitoring models for various pediatric ocular diseases, thereby providing support for understanding their pathogenesis and optimizing clinical intervention strategies.

## CONCLUSION

In conclusion, although current OCTA imaging cannot detect dynamic, real-time leakage and has limited ability to capture far-peripheral images, it does provide a rapid, safe, and non-invasive method to track and monitor disease progression, which may be particularly beneficial for pediatric patients. Cross-sectional information and quantitative vascular parameters from the retinas of healthy and diseased children may contribute to diagnosis, follow-up, and assessment of treatment response.

Although OCTA is not yet the standard method for assessing pediatric myopia and amblyopia, it provides both qualitative and quantitative information on retinal microvasculature and anatomy, allowing for objective tracking of changes across visits. Research on OCTA has identified many potential applications, but their translation into routine clinical practice remains uncertain. This review argues that OCTA can reveal various clinical features of myopia and amblyopia in children, which can guide future investigations. Favorable visual outcomes in many pediatric eye diseases depend on early detection, close monitoring of progression, and assessment of treatment response; therefore, sensitive measures that support these goals are invaluable. With advances in OCTA hardware and software, the establishment of standardized databases, and integration with artificial intelligence, OCTA has the potential to become a useful tool for ophthalmologists evaluating pediatric myopia and amblyopia.

## Figures and Tables

**Fig. (1):**
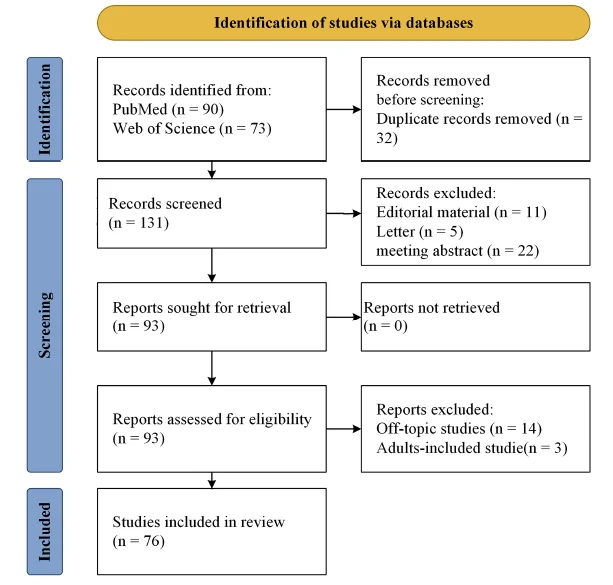
Literature screening flow chart.

**Fig. (2):**
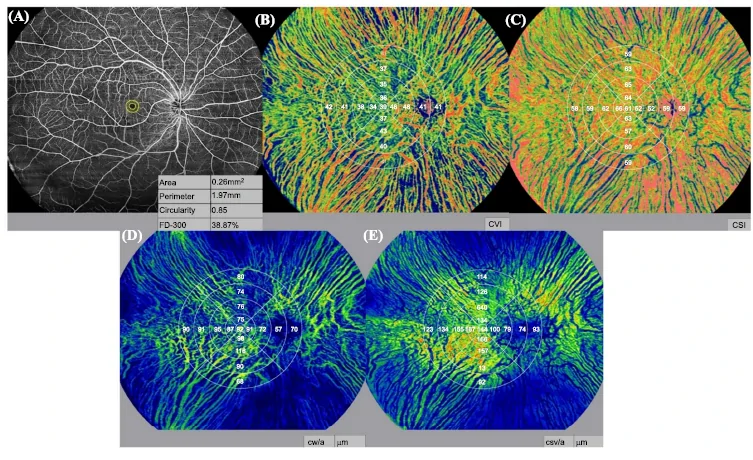
Representative swept-source OCTA images. (**A**) Foveal avascular zone (FAZ) with related measurements (area, perimeter, circularity). (**B**) Choroidal vascularity index (CVI). (**C**) Choroidal stromal index (CSI). (**D**) Choroidal vascular volume (CVV). (**E**) Choroidal stromal volume (CSV). Automated 1-, 3-, and 6-mm ETDRS rings are shown as reference in each panel.Reprinted from BMC Ophthalmology, Volume 26, Article number 76, Serap Karaca, Ahmad Kunbaz, Osman Kizilay, Bilge Batu Oto, Gokhan Celik, Structural and microvascular changes in anisometropic amblyopia: a comparative analysis of swept-source OCTA, Pages 3, Copyright (2026), licensed under CC BY-NC-ND 4.0 [19].

**Fig. (3):**
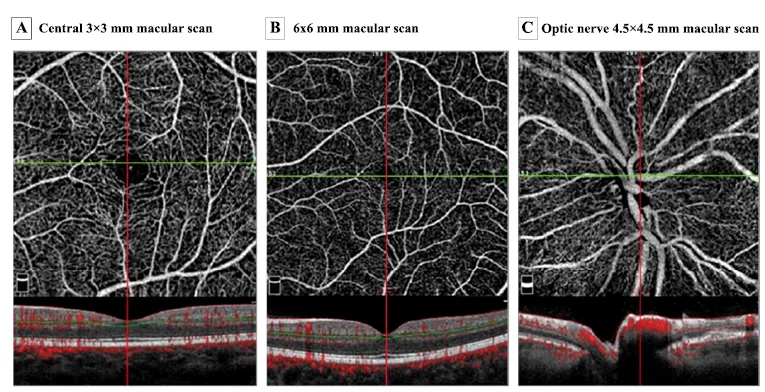
Optical coherence tomographic angiography scans.Reprinted from JAMA Ophthalmology, Volume 135, Issue 10, Marcela Lonngi, Federico G. Velez, Irena Tsui, Juan Pablo Davila, Mansour Rahimi, Clarissa Chan, David Sarraf, Joseph L. Demer, Stacy L. Pineles, Spectral-Domain Optical Coherence Tomographic Angiography in Children With Amblyopia, Pages 1088, Copyright (2017), with permission from American Medical Association [27].

**Fig. (4):**
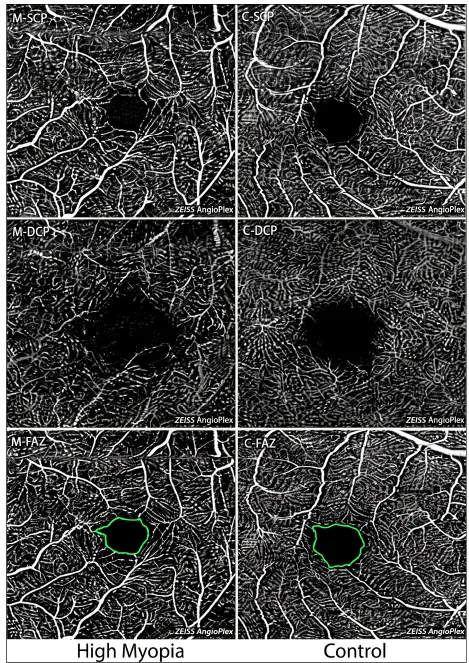
Optical coherence tomography angiography images of typical high myopia and control eyes. The OCTA images were segmented at the SCP (top) and the DCP (middle). The green borders outline the FAZ at the SCP (bottom). VD decreased in the SCP but remained unchanged in the DCP, FAZ remained unchanged in high myopia. Reprinted from Ophthalmic Surgery, Lasers and Imaging Retina, Volume 49, Issue 12, Yunxia Leng, Emily K. Tam, Khalil Ghasemi Falavarjani, and Irena Tsui, Effect of Age and Myopia on Retinal Microvasculature, Pages 928, Copyright (2018), with permission from SLACK Incorporated [29].

**Fig. (5):**
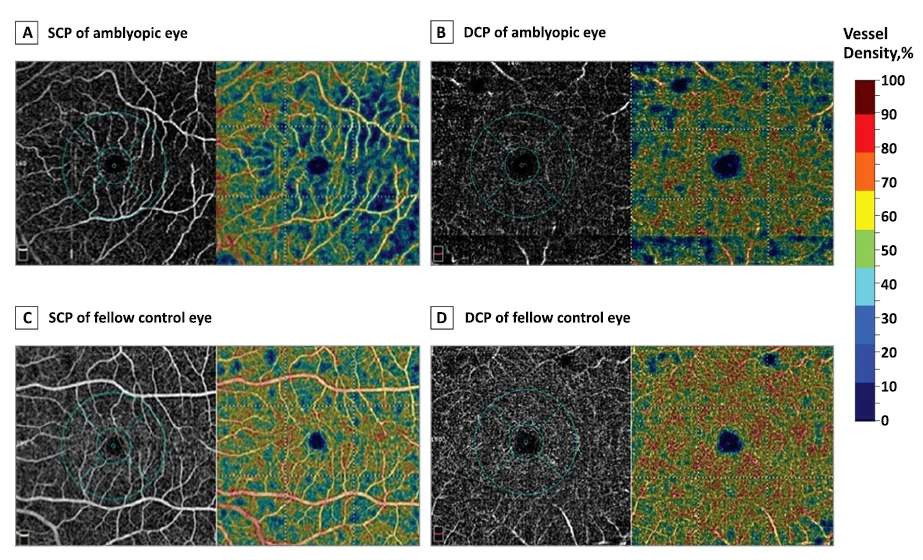
Optical coherence tomography angiography images of typical amblyopic and control eyes. The OCTA images were segmented at the SCP (**A** and **C**) and the DCP (**B** and **D**). In the perfusion density images (images on the right), reduced vessel density was represented by bluer hues. VD decreased in the SCP and DCP in amblyopic eyes.Reprinted from JAMA Ophthalmology, Volume 135, Issue 10, Marcela Lonngi, Federico G. Velez, Irena Tsui, Juan Pablo Davila, Mansour Rahimi, Clarissa Chan, David Sarraf, Joseph L. Demer, Stacy L. Pineles, Spectral-Domain Optical Coherence Tomographic Angiography in Children With Amblyopia, Pages 1088, Copyright (2017), with permission from American Medical Association [27].

**Table 1:** Structured summary of OCTA findings in pediatric myopia.

**Vascular Region/Layer**	**Main OCTA Metrics**	**Overall Reported Tendency**	**Clinical Interpretation**
SCP	Macular VD, foveal/parafoveal VD	VD often reduced with increasing myopia severity; however, some studies show no significant change	May reflect early inner retinal microvascular remodeling associated with axial elongation, but results are influenced by scan area, age, and device differences, *etc*
DCP	Macular VD, perifoveal VD	Findings are mixed; several studies report reduced DCP VD in high myopia	Should be interpreted cautiously, and is affected by projection artifact, segmentation method, *etc*
FAZ	Area, circularity, perimeter, volume	Inconsistent: some studies report enlargement, whereas others show no significant change	Differences may be driven by AL, age, myopia severity, and device software
Choriocapillaris	Flow area, flow voids, CCFD	Reduced flow features or increased flow deficits	Suggests involvement of the choriocapillaris in myopia development and progression.
Choroid	CT, CVI, CVV, CSV	Thinner choroid and reduced choroidal vascular parameters	May represent AL elongation-related choroidal remodeling
ONH	IVD, PVD	PVD decreased with increasing AL, especially in temporal regions, while IVD remained relatively stable	Supports broader vascular involvement beyond the macula in high myopia

**Table 2:** Structured summary of OCTA findings in pediatric amblyopia.

**Vascular Region/Layer**	**Main OCTA Metrics**	**Overall Reported Tendency**	**Clinical Interpretation and Limitations**
SCP	Macular VD, foveal/parafoveal VD	Most studies report reduced SCP VD in amblyopic eyes, but some subtype studies show no significant change	SCP reduction may reflect abnormal inner retinal vascular development or reduced metabolic demand caused by insufficient visual stimulation. Results are affected by subtype, scan size, device, and control selection
DCP	Macular VD, foveal/parafoveal VD	DCP VD is often reduced in amblyopic eyes; foveal DCP VD may increase after patching treatment	DCP may be sensitive to amblyopia-related microvascular alteration and treatment response, but results may be influenced by projection artifacts and segmentation differences.
FAZ	Area, perimeter, circularity, morphology	FAZ findings were inconsistent. Some studies reported larger or smaller FAZ, while others found no significant difference.	FAZ is less stable than VD as a biomarker and is influenced by age, ethnicity, refractive status, axial length, and magnification correction
Choriocapillaris	Choriocapillaris flow area	Evidence is limited. Some hyperopic anisometropic amblyopia studies suggest increased Choriocapillaris flow area	Current clinical value remains exploratory
Choroid	CT, CVI, CVV, CSV, 3D-CVI/3D-CVV	CT is often increased in hyperopic amblyopic eyes, but CVI/CVV/CSV findings are inconsistent	Choroidal changes suggest possible deeper vascular involvement, but results are confounded by hyperopia, AL, age, amblyopia subtype, and 2D/3D analysis methods
ONH	Optic disc VD, peripapillary VD, radial peripapillary capillary VD	Evidence is limited and inconsistent. Some studies show no optic disc VD difference, whereas others report reduced radial peripapillary VD	ONH parameters may reflect broader retinal ganglion cell or neurovascular involvement, but evidence is weaker than macular SCP/DCP findings

## LIST OF ABBREVIATIONS

OCTA: Optical coherence tomographic angiography
FAZ: Foveal avascular zone
VD: Vascular density
RT: Retinal thickness
CT: Choroidal thickness
SCP: Superficial capillary plexus
DCP: Deep capillary plexus
CVI: Choroidal vascularity index
CVV: Choroidal vascular volume
CCFD: Choriocapillary flow deficit
CSI: Choroidal stromal index
CSV: Choroidal stromal volume
CC: Choriocapillaris flow area
AL: Axial length
ONH: Optic nerve head
IVD: Intrapapillary vascular density
PVD: Peripapillary vascular density
LLLT: Low-level red-light therapy
